# The Late Meeting of the Texas State Medical Association

**Published:** 1895-05

**Authors:** 


### Society Notes.

### The Late Meeting of the Texas State Medical Association.

It has been customary with this JOURNAL for years to publish a synopsis of the proceedings of the annual meeting of the State Medical Association, and the JOURNAL expected to give a full report of the late meeting in this issue; but we failed to receive a copy of the minutes from the stenographer, and having taken no written notes, we have to rely upon our memory, and partly upon the very unsatisfactory and disconnected report in the *Dallas News*.

The 27th annual meeting was held in the fourth story of the City Hall; up four flights of steps ye delegates and members had to climb—old and young; there was no elevator, and we can vouch for the assertion we heard more than one man make,— "it makes a fellow tired."

The meeting was looked forward to for months, with anxiety and interest. It was designed to be a harmony meeting, a final reconciliation of the sections, north and south, between which there was a split some years ago, it will be remembered,—they split on the subject of religion; or, rather, because of a criticism of the JOURNAL (whose editor was at that time Secretary), strongly deprecating the introduction of religion into the President's address and in the discussion,—protesting against a repetition of it, as dangerous to the peace, and foreign to the objects of the Association. Exceptions were taken to our remarks. Feeling was engendered, and the northern delegates largely withdrew.

The editor resigned the position of secretary in the interest of peace, and worked for a restoration; harmony was restored; the last disturbing element had been eliminated, and this meeting was to cement the new union.

In one sense it was a failure, we regret to say. The north showed up strongly, thus evincing a willingness to forget and forgive, and come back into the fold; but the south was very scantily represented, there being but two representatives present from Houston, two from San Antonio, three from Galveston and five from Austin. It was not due, however, to any disaffection on the old score, but was a coincidence, perhaps; or due to hard times,—or, was it due to an inherent defect in the organization of the Association? Whatever may have been the cause—the attendance was slim, not over one hundred and fifty being in attendance, and the last day the attendance upon the installation of the new officers was reduced to about thirty.

The President called attention to the steady yearly decadence of the Association, and in his "address and recommendations" suggested the appointment of a committee to report upon the best means of arresting it; of removing the causes, if they can be ascertained—and of promoting a new growth, with the properties in it, of adhesion; some plan by which physicians who have never been members, may be brought into the fold; others, who have been in, and who have dropped out, brought back,—and all made to stick.

The JOURNAL, it will be remembered, several times called attention to the steady decline in membership, and has as often expressed its opinion of the cause. We have advocated a change in the organization, and have insisted upon a reduction of the cost of membership. But the consensus of opinion appears to be adverse to converting the Association into an annual convention of delegates from local societies,—to be thoroughly organized—as advised by us,—preferring to adhere to the original plan.

We give below the report of the committee on the subject of arresting the downward tendency, etc., and it will be seen that amongst their recommendations is a reduction of cost of membership, and economy in disbursements; a less costly volume, and less compensation to the secretary, who now has the aid of a stenographer.

The committee, through its chairman, Dr. Wooten, reported as follows:

In our judgment the first and fundamental requisite for reviving and extending the usefulness and efficiency of the organization is an intelligent and practical understanding of its objects and reason for existence. The purpose of the State Medical Association is, to promote scientific research and investigation in the profession, with a view to the practical application of the great principles of medical science to the daily needs of humanity and the current practice of the physician. To that end its meetings should be devoted to terse, pertinent and vigorous discussion of recent discoveries, approved experience and well-attested facts in the domain of medicine and its allied branches. It was never intended that the time and attention of the members should be consumed in idle speculations, imaginary exploits or vapid generalizations. Neither was it contemplated that essential and fundamental principles should be reiterated and renewed at every session, and in prolix and prosy dissertations on elementary topics with which every intelligent and properly-equipped doctor is presumed to be already familiar. The social pleasures of these annual meetings are incidental to these main objects, and it is safe to say that even they will be greatly enhanced by a rigid attention to the scientific aims of the organization.

Having in mind these practical purposes of the organization, and believing that their accomplishment is the surest way to increase the attendance and enlist the interest of the profession of the State at large, we recommend, or suggest, the following methods of attaining them:

1. That no paper intended for presentation to the Association or any of its sections, should exceed a length that will permit of its being read within twenty minutes. All papers should be presented to the chairman of each section a sufficient time before the meeting of the Association to permit of its examination by him and the secretary of the section, and it shall be the duty of the chairman and the secretary of each section to examine all papers thus submitted, to eliminate all irrelevant, improper and useless matter therefrom, and if it exceeds the above length, to cut it down by striking out all but the most important and valuable matter, so as to bring it within the specified limit. The paper, if deemed worthy of presentation to the Association, shall then be returned to its author for revision, in accordance with the action of the chairman and secretary of the section, and also for the author to prepare a brief and comprehensive synopsis of its contents and purport. All papers and discussions shall be strictly scientific, and shall not involve or refer to religious or other foreign subjects of debate.

2. All papers presented the Association, and its several sections, shall be presented simply by reading the synopsis prepared as above suggested; and unless the Association, by a vote, shall call for its reading in full, it shall be discussed and referred to the proper committee by its synoptical contents alone, and not by reading it to the Association.

3. In the discussions of all papers, no member shall be permitted to speak more than once to the same subject, and all speeches shall be limited to five minutes in length, which may be extended to ten minutes by vote of the Association.

4. The publication committee should exercise a wise but rigid discretion in the publication of papers presented to the Association, whether by synopsis or in full, and should not publish any but papers of practical and permanent value to the profession. The discretion thus vested shall not be taken away from the publication committee by a vote of the house of less than four-fifths majoiity.

5. The membership fee for initiation should be reduced to $2.50, and the annual dues to $2.50, so as to encourage young men and new comers to join, and to retain their connection with the Association. It is also believed that the expenses of publishing the Transactions can be reduced, and that the salary now paid the Publishing Committee can be reduced, or partly divided with the Secretary, whose labors are much lessened by the stenographers.

6. The prolific and pernicious source of weakness in the Association heretofore, in which it is not different from many others, has been the prevalence of self-seeking in the disposition of the principal offices of the organization. To the end that this may be effectually stopped, we recommend that all nominations for the officers of the Association shall be made by a nominating committee, who shall be required to nominate no man for any office who has been or is an aspirant for it, or who has directly or indirectly solicited the same at the hands of the committee, or any member of it.

7. Another source of annoyance, amounting to a positive hindrance to the success of our meetings, is the inability to hear the papers and addresses that are read before us. We suggest that whenever it is apparent that any person can not make himself heard in the delivery of a paper before the body, it shall be the duty of the President, or chairman of a section then in the chair, of his own motion, or upon the suggestion of the members, to have said paper or address read in an audible and intelligent manner by the Secretary, or by some member of the Association competent to do so; and if necessary, a reading clerk shall be appointed at each meeting for that purpose.

8. We recommend that the Association do encourage district and county associations, as valuable adjuncts to this organization.

The meeting was a success, if judged, not by numbers, but by the quantity and quality of work done in Sections; there were many able and valuable papers read, all, or most of which will appear in the volume of Transactions about July or August, and from time to time in the JOURNAL. We give, this issue, two of the papers; the very strong and scholarly paper by Dr. Wallace, which created so much excitement,—and, we fear, feeling, in as much as the Publishing Committee has since refused to publish it in the yearly Transactions; and one by Dr. Bermingham, the well known specialist of New York, contributed to the JOURNAL by the author's especial request, after having been read by the Secretary. Dr. Bermingham was not present.

Some of the papers, however, were not so good, as was to be expected. There was one paper read of which the *Dallas News* says:

Dr. J. W. Hunter, of Waco, read a paper on "Asthma—Its Causation and Treatment, with Report of Cases," after finishing which he invited ample discussion of the subject. Several members had discussed the subject casually, when Dr. Yates arose and opposed the reception of the paper by the Association for publication, on the ground that it advertised proprietary remedies. This brought Dr. Hunter to his feet. He said that he did not read the paper as an advertisement of proprietary medicines, but for the benefit of medical science. He said that he was an old member of the Association, and that he had a right to read anything he wanted to. A motion was made, however, to exclude the paper from publication, which received seconds from all parts of the house. The motion was carried.

Dr. Hunter is traveling agent for a firm who manufacture a preparation "*good for asthma.*"

There were present a number of distinguished medical men from abroad. They were treated with marked courtesy, and we feel sure can have no cause to regret having been with us on that occasion. Their presence was a compliment to the Association. It shows that the work heretofore done by the Association has attracted attention. That is one reason why we deplore the slim attendance. Amongst those present were Prof. Richard Douglas, of the Vanderbilt Medical College; Prof. Landon Carter Gray, of New York; Dr. J. M. Kellar, of Hot Springs, too well known to require any introduction anywhere in these United States where medicine is known or talked; and Dr. G. W. Wells, the able and distinguished medical director of the Manhattan Life Insurance Co. of New York, and editor of the sprightly *Medical Examiner*, a monthly journal devoted to the insurance examination interest.

The Association did a graceful thing in transferring from the active to the honorary members list the honorable and venerable Dr. Samuel Hollingsworth Stout, A. M., M. D., LL. D., the distinguished Medical Director of all the Hospitals of the Southern Confederacy, and Dr. Daniel DuPre, another old Confederate surgeon. Drs. Gray and Douglas were also made honorary members.

It would seem invidious to select for special mention and commendation any paper or papers which were read, where so many were good; but we will be pardoned, we hope, for referring especially to that by Dr. J. T. Wilson, of Sherman, on "The Modern Methods of Educating Girls." It was regarded by many as a brilliant paper, though it read simple, plain truths. Dr. Wilson vigorously assailed the present method of conducting the schools, colleges and educational institutions of the country. He referred to the harmful or want of proper clothing and food of girls, and the want of care for their condition by their parents. He said that the nerotic girlhood of the period was due to the absolute neglect of mothers. Dr. Wilson, when asked for the paper for publication, refused to give it to the public.

Dr. Landon Carter Gray said, it was a splenpid paper, and that Dr. Wilson deserved great credit for his courage in making public matters which every medical man knows have existed, but which few have had the courage to put into definite assertions, or to publicly announce.

Dr. J. O. McReynolds, of Dallas, welcomed the Association in a handsome speech, as did his Honor, Mayor Holland. The Committee of Arrangements left nothing undone, not only to make the visitors comfortable and happy, but to assure them of a cordial welcome. There had been some talk of the cold shoulder being possibly turned towards us; but there could hardly be conceived a more cordial, pleasant greeting than that given the visitors. The best men of the profession were in and out the hall—participating more or less in the proceedings—as their duties to patient spermitted. Drs. E. L. Thompson, S. Eagon, J. M. Pace, H. K. Leake, H. N. Mosely, R. H. Chillon, Armstrong, McReynolds, Letcher, Dupre, Stout, were in and out greeting every one with a smile, a handshake and a welcome.

Dr. J. S. Letcher, of Dallas, in the absence of the appointed chairman, presided over the Section on Practice, and made an able and dignified Chairman.

Elegant receptions were given the guests at the residences of Drs. Thompson, Leake, Chilton, Eagon, and Letcher; and a royal Reception and Banquet was given them at the Oriental Hotel.

The Oriental Hotel is a magnificent establishment, perfect in every detail for comfort and convenience,--extremely swell. It was beautifully decorated with flowers and ornamental plants in honor of the occasion. The only objection this scribe found to it was the *menu* in French! What an absurdity! and bad French at that; spliced out with plain English, whenever the "Chef," or whoever prepared it, stumbled on a word the French for which he did not know. Give Texas doctors good wholesome, plain food, called by its name in plain United States, and they are "at home."

We missed our old stand-by, Dr. W. P. Powell, of Willis, as did every member present; for it is the first meeting he has failed to attend in twelve years, to our certain knowledge.

Of course, notice was taken of the death of our grand old surgeon, Dr. Cupples, which occurred only four days previous to the meeting. No one present was in possession of sufficient data in his life's history to there and then prepare such resolutions as his great and honored name, and the place he occupied in the Association and in the affections of the members, demanded. The committee, of which Dr. Wooten is chairman, we believe, will prepare a sketch of his life, and resolutions of respect, etc., and have it appear in the local papers first, and then in the Transactions.

Another excellent paper was that of Prof. Clopton, on Physical Culture. It should be extensively circulated and read, for the Professor is an authority on physiology, being teacher of that branch in the Texas Medical College, and one of the Association's ablest speakers and writers.

And the handsome, dashing young Scotch-Irish favorite, Preston C. Coleman, of *away out* west, was unanimously elected President! Told you so! It will be remembered the Red-back nominated him for President just a year ago—in our last May number, just after the election of McLaughlin last meeting; and it is with much pleasure and satisfaction that the JOURNAL presents herewith his photo and biography.

Dr. T. J. Wagley, of Cleburne, another talented and dashing young doctor—young—i. e., compared to some of us—old fellows—ahem!—was elected 1st Vice-President; Dr. West was re-elected Secretary, and (of course) Dr. J. Larendon was re-elected Treasurer (for the twenty-second consecutive year).

Dr. J. W. McLaughlin, the retiring President, made a dignified and able presiding officer. His address, which occcupied an hour and thirty minutes, was listened to (it was delivered on the second night) with close attention by a splendid audience of ladies and gentlemen. It was pronounced a most learned effort. The subject was the "Dynamics of Medicine," or words to that effect.

Our fine looking colleague, Bennett, of the *Texas Sanitarian*, was chosen as President of, and presided over the Nominating Committee. He doesn't speak to anything less than a Major-General now.

Dr. McReynolds in his paper on Ophthalmia Neonatorum said that 15 per cent. of blindness was due to ophthalmia neonatorum. This paper is most important, especially in consideration of the fact that the late lamented 24th Texas legislature refused to pass a bill to prevent blindness by requiring infantile sore eyes to be attended to at once. It will be remembered that in our last we spoke of an old fellow on the committee on public health, last legislature, who killed the bill by saying, "Mother's milk will cure all the baby sore eyes." Dr. McR. despairs of legislative aid, therefore, and says:

"Now, by what means can we secure the enactment of appropriate laws looking to this urgent need? Several States have already statutes relating to this important subject. But with regard to a disease so extensively found all over the country, so rapidly destructive when not met with intelligent treament at the threshold of its career, and so easily controlled by methods at our command, the broadest legislation is the best. And it would be most desirable to have a law on this question enacted by the national congress, and if this at present is impracticable, try the State legislature, and if this distinguished body has no time to consider the public health, happiness and economy, then we can begin with the plain and humble spirit of charity, at home. And I propose, yea, I do now, at this very moment, petition the city council of Dallas to take such action with regard to this disease as will result in its complete prevention in our city, or successful issue when once developed. And I advise that an ordinance be enacted similar to the following: First, that all obstetrical attendants, professional and non-professional, shall be required to carry into execution those measures which will protect the infant against ophthalmia neonatorum. Second, that when the disease has become established in any case, efficient medical service shall be procured without delay."

It is most gratifying to observe that many new members were added to the roll at Dallas. We have not been able to get a complete list, but amongst the members were: Drs. T. G. Duncan, of Victoria ; E. P. Davis, of Houston; E. J. Neathery, of Van Alstyne; A. T. Perkins, of Gainesville; R. R. Walker, of Paris; E. N. Camp, of Lewisville; W. P. Lee, of Cisco; F. A. Young, of Navasota; W. F. Cole, of Waco; Joe Myers, of Honey Grove; R. G. Powell, of Lewisville; Joe D. Becton, of McKinney; C. A. Shultz, of Alvarado; H. W. Wandless, of Dallas; W. H. Moore, of Weesatche, F. C. Todd, of Fort Worth; W. M. Moore, of Paris; J. A. Lovell, of Abbott, J. W. Miller, of Hillsboro; J. W. Hamilton, of Lampasas; J. A. Jones, of Ferris; W. J. Lane, of Dallas; M. D. Edmonson, of Dallas: Thomas W. Florer, of Waxahachie; F. P. Peyton, of Dallas; I. M. Cline, of Galveston; T. T. Jackson, of Iredell; G. M. Hackler, of Ennis; G. C. Head, of Grand View; S. D. Naylor, of Stephenville, P. S. Gilbert, of Oak Cliff and J. M. Coble, of Dallas.

Dr. Landon Carter Gray contributed a paper on the "Value of Knowledge to the General Practioner of Nervous and Mental Disease." Dr. J. B. S. Holmes, of Atlanta, Ga., also contributed a paper, which after being read and voted to be published in the Transactions, was handed to the TEXAS MEDICAL JOURNAL for publication. It will appear in our next issue.

Our Dr. S. E. Hudson is absent, and will leave yours truly the bag to hold, so to speak—an entire month. He left on May 3rd, for Baltimore, to attend the meeting of the American Medical Association, the Editors' meeting, the Medical Publishers' meeting, etc., at Baltimore; thence he went to New York. Here he will put in a month at the polyclinic and the hospitals, and then take a run up Niagara and other points, having a good time generally, we hope. Mrs. Hudson accompanies the doctor. Treat him well, all ye friends of the "Red-Back," and of Daniel individually; he's worthy of it; he's a better man than "we," but not half so good looking.

The Texas State Medical Association will meet next year at Fort Worth, last Tuesday in April. Hope to meet you all there.

---

The general appropriation bill passed by the legislature just adjourned, gives the Quarantine Department $33,000 a year, for all purposes. Senate bill 130, which was finally passed, reduced the salary of the coast officers very heavily, to-wit: from $300 a month to $150, except at Galveston; the officer at that port is to receive $200, and will most likely be kept on duty throughout the year. The other coast officers are on duty usually only during the quarantine season—May to November. On the Rio Grande, there are three inspection stations, Laredo, Eagle Pass and El Paso. The instector's pay has been fixed at $150 a month (they formerly received $5 a day, and were on duty the entire year, on account of the prevalence of small-pox in Mexico). The State Health Officer suffers a reduction in his salary also, his pay being fixed at $2500 a year, just a fraction more than the officer at Galveston receives.

The mutilation of this bill in the senate was the result of a misapprehension of facts as to the relative duties of the officers, notwithstanding everything had been fully explained to the committee having charge of it. The senators evidently intended to make the pay of quarantine officers uniform, and hence it was fixed at $150, with exception noted. They did not seem to understand that the inspectors are at home,—are on duty all the time, and only have to inspect trains, and other incoming travel. They can carry on their practice meantime. They were being paid, under the old law, $5 per day, and as the new bill gives them $150 a month, it is practically the same thing. But the quarantine officers on the coast are taken from their homes from May to November. Their practice, of course, is neglected, and likely to go to the bow-wows, *or some other fellow*, during their absence. They are isolated, perhaps on a sand bank away out in the gulf, or on some dangerous point, or pass, exposed to the heat, to danger of infection, to storms; live hard and lead a lonely life, and suffer many hardships. They receive pay only for six months,—sometimes five months,—and were paid $10 a day; they are now to get only $150 per month.

The new law, senate bill 130, was made in accordance with the governor's suggestions, and to meet the cut in the appropriation. It provided $200 a month for the coast, and $150 a month for the border officers; but the senators, as stated, thought they should all fare alike, and hence the reduction in the pay of the coast men. It will be seen that while the border officers, who have a comparatively easy berth, and, if on duty all the year, receive $1800, the coast officers are only paid $900 for the six months they are on duty. It was the governor's wishes that the former receive only $1000, and the latter $1200, presumably (the latter) for six, and the former for twelve month' service. The bill as prepared and introduced, would have very nearly met his views, as it provided for $200 a month for six months on he coast, and $100 a month for a year (probably) on the border.

